# Non-curative care options for patients with advanced-stage head and neck cancer: Current state of the science and future opportunities

**DOI:** 10.1017/S1478951524002049

**Published:** 2025-02-17

**Authors:** Melissa C. White, Julia E. Canick, Yvonne M. Mowery, Daniel J. Rocke, Katherine Ramos, Nosayaba Osazuwa-Peters

**Affiliations:** 1 School of Medicine, Duke University, Durham, NC, USA;; 2 Department of Otolaryngology - Head and Neck Surgery, NYU Langone Medical Center, New York, NY, USA;; 3 Department of Radiation Oncology, Duke University Medical Center, Durham, NC, USA;; 4 Duke Cancer Institute, Durham, NC, USA;; 5 Department of Head and Neck Surgery & Communication Sciences, Duke University School of Medicine, Durham, NC, USA;; 6 Durham Center of Innovation to Accelerate Discover and Practice Transformation, Durham Veterans Affairs Medical Center, Durham, NC, USA;; 7 Department of Population Health Sciences, School of Medicine, Duke University, Durham, NC, USA;; 8 Department of Psychiatry and Behavioral Sciences, Duke University, Durham, NC, USA;; 9 Department of Medicine Geriatrics, Duke University, Durham, NC, USA; 10 Center for the Study of Aging and Human Development, Duke University School of Medicine, Durham, NC, USA

**Keywords:** Non-curative intent, palliative care, end-of-life (EOL) care, home and hospice care, a good death, head and neck cancer (HNC)

## Abstract

**Objectives.:**

Head and neck cancer (HNC) often requires complex management and care. While the primary goal of treatment is curative, some advanced cases require consideration of non-curative pathways to optimize patients’ quality of life (QOL) and survival. This narrative review describes important aspects of palliative care and highlights strategies for employing these non-curative options in HNC.

**Methods.:**

We identified peer-reviewed articles on the state of palliative care in HNC and its implementation. We searched for articles using terms including “palliative care,” “non-curative care,” “comfort care,” “head and neck cancer,” and “head and neck squamous cell carcinoma.”

**Results.:**

HNC is associated with a high disease burden; patients report high levels of pain, and both disease and treatment often compromise ability to carry out activities of daily living. There exist several non-curative routes of treatment, including palliation of symptoms, acute end-of-life (EOL) care, and hospice and home care. These care options provide comfort and optimize QOL of patients. Unfortunately, non-curative care could be misconstrued as withdrawal of treatment, or the provider team “giving up” on patient; these misconception can discourage patients from embracing palliative measures designed to alleviate symptom burden. Proper physician–patient communication, normalization, and early incorporation of these non-curative strategies into mainstream treatment could potentially ease patient concerns, and, eventually in EOL cases, help patients achieve dignified deaths.

**Significance of results.:**

Patients with HNC have unique palliative care needs due to their complex treatment and symptom burden. Early incorporation of non-curative plans such as palliative care alongside active treatment could help reduce symptom burden. Clinicians should strive to build trusting relationships with patients with HNC and effectively communicate with them about palliative care options. Guidelines that include such recommendations can help physicians regularly introduce palliation into the realm of active HNC treatment for advanced/incurable disease.

## Introduction

Head and neck cancers (HNCs) are particularly painful and morbid cancers (Cramer et al. 2018) that account for over 400,000 deaths annually worldwide (Observatory). HNC patients increasingly present with late-stage disease (Thompson-Harvey et al. 2020), which can necessitate aggressive, multimodal therapy (Patel and Brennan 2012) and often carries a poor overall prognosis (De Graeff et al. 2001). HNC and treatment-related toxicities have a disproportionate impact on quality of life (QOL) and patients’ abilities to carry out several fundamental activities, including breathing, chewing, and speaking (Zeller 2006). In addition, HNC patients often have comorbid physical and psychiatric conditions that increase the distress associated with their diagnosis and treatment (Osazuwa-Peters et al. 2021; Penner 2009).

Management of HNC usually prioritizes aggressive treatment using a combination of surgery, radiation, and chemotherapy; however, an equally essential consideration in the management of these complex patients is the use of non-curative pathways – namely symptom palliation, hospice, and end-of-life (EOL) care. Recent studies have suggested that the early integration of palliative care services, potentially alongside active disease treatment, into an advanced cancer patient’s regimen has many benefits (Ferrell et al. 2017) including improved QOL, better survival rates, fewer mood symptoms, and less aggressive EOL care (Chen et al. 2019; Chou et al. 2021; Greer et al. 2012; Temel et al. 2010). Despite the advantages of incorporating palliative care into cancer therapy, this type of service is often underutilized (Obermeyer et al. 2015), in part because it is often not offered until later in a patient’s course of treatment (Gidwani et al. 2016; Schenker et al. 2016) in both general cancer care and in HNC.

The aims of the present review are to describe non-curative treatment modalities for HNC patients who experience disproportionately high disease burden and to consider qualities that might help them achieve satisfaction with their care, improved QOL, and, when treatment is not curative, the most dignified deaths possible. An understanding of these options is essential to helping patients with advanced stage HNC and their caregivers optimize end-stage cancer care.

## Observations

### HNC-specific disease burden

HNC patients use non-curative modalities less frequently than do other cancer patients (Bauman and El-Jawahri 2021), even though many of these individuals have high symptom burdens and other comorbidities, which increase their chances of dying in the hospital rather than at home or in hospice (Stephens et al. 2020). No universally adopted plans or guidelines exist for palliation and hospice care for cancer patients, including for HNC patients who would likely benefit substantially from such care (Catriona Rachel Mayland et al. 2021a).

Symptoms related to both disease and treatment toxicities are commonly physically and psychologically distressing for HNC patients and their caretakers. HNC affects basic human functions, including speaking, eating, and breathing (Schenker et al. 2016), and causes diminished senses of taste, hearing, and smell. Tumors in the head and neck can also cause airway obstruction and dysphagia (Chan et al. 2013; Schenker et al. 2016), which can lead to further complications including aspiration pneumonia, tracheotomy, and malnutrition (Bonomo et al. 2019) and contribute to recurrent hospitalizations and increased morbidity.

Both HNC and its treatment are associated with pain, especially if the surgical treatment involves neck dissection or radiation (Brook 2020; Gostian et al. 2021; Terrell et al. 1997). In fact, while discomfort is common across cancer sites in general, 70% of HNC patients report pain – the highest prevalence of all cancer patients (Van den Beuken-van Everdingen et al. 2007), possibly due to the rich somatic innervations of the region and confinement of several anatomical structures to a small space (Macfarlane et al. 2012). Further, surgical resection of late-stage cancer often requires free flap reconstruction, which causes pain to regions outside of the head and neck (Rogers et al. 2003). Radiotherapy (Havard et al. 2021) and chemotherapy (Dugué et al. 2022) are other significant sources of pain, primarily due to mucositis and dermatitis. In addition, HNC commonly presents in older patients, many of whom have long-standing histories of tobacco or alcohol use, giving rise to comorbid conditions that often aggravate their pain (Bonomo et al. 2019).

Furthermore, HNC patients are subject to psychological distress as they cope with their illness and treatment-related toxicities. In addition to causing pain, HNC tumors and surgeries commonly cause disfigurement, which can be upsetting for patients and their loved ones (Frampton 2001). This may be associated with some level of shame, stigma, and self-blame (Schenker et al. 2016), especially given these cancers’ associations with tobacco, alcohol use, and HPV (a sexually transmitted disease) (Dodd et al. 2016; Kissane et al. 2013). This guilt, along with a perceived loss of functional independence and diminished ability to speak or swallow, can cause patients to feel isolated and restricted in their social activities (Penner 2009). These patients have a higher reported prevalence of anxiety and depression when compared with many other cancer patients – up to 57% in oropharyngeal cancer patients (Massie 2004; Zabora et al. 2001).

### Palliative care

The World Health Organization (WHO) defines palliative care as “an approach that improves the quality of life of patients (adults and children) and their families who are facing problems associated with life-threatening illness” (Organization WH 2020). Within oncology, this translates to the alleviation and management of cancer-related symptoms that focuses on QOL (Saeed et al. 2018). Importantly, this type of care is not mutually exclusive with care aimed toward curing the underlying disease (Schlick and Bentrem 2019). Though palliative measures are helpful for patients with HNC and patients in general, there exist several barriers to their inclusion in the standard of care.

The idea of palliative care has undergone a paradigm shift over the last several years; before recent advances in the conceptualization of such treatment, the commencement of palliation was often viewed as the treatment team “giving up” on the patient (van den Heuvell et al. 2017). Studies have noted the importance of fully involving a palliative care team in all advanced-stage cancer treatment (Chen et al. 2019), and this care can be concurrent with and complementary to active treatment of disease (Ferrell et al. 2017). However, this involvement is not yet considered to be part of the standard of care for HNC. Notably, the American Society of Clinical Oncology (ASCO) Clinical Practice Guidelines for integration of palliative care into standard oncology care does not mention HNC (Ferrell et al. 2017). Additionally, social factors and common misconceptions might contribute to patient reluctance to pursue palliative care. In a recent national survey, 42% of adult participants responded that the idea of palliative care automatically made them think of death (Adjei Boakye et al. 2020). Erroneous beliefs about comfort-based care, such as the notion that palliation is reserved for individuals with life expectancies under 6 months (in actuality, this is true only of hospice) and the conflation of palliation with hospice, might contribute to a general hesitancy to engage in palliative measures (McIlfatrick et al. 2021). A patient with late-stage cancer that is not responding to multiple treatment modalities might find a sudden introduction of non-curative care to be a sign that his or her care team is no longer interested in exploring other options; the early integration of palliative care into treatment regimens may help assure patients that their healthcare providers will not be “cutting corners” by not pursuing more aggressive treatments during later stages of their diseases. This is especially relevant in HNC as patients may face significant decline toward the end of life (van den Heuvell et al. 2017). Furthermore, socioeconomic factors, including race, ethnicity, poverty, immigration status, and insurance coverage all might contribute to a lack of palliative care utilization (Ferrell et al. 2017; Ramsey et al. 2021; Sullivan et al. 2021). Palliative care is often inadequately covered by insurance or by Medicaid (Ollove 2017; Sullivan et al. 2021), providing yet another barrier to this management.

In addition to some patients’ reluctance and erroneous perception of palliative care, there are other access to care factors impacting routine implementation of palliative care in advance HNC care. While most patients with HNC have been found to have palliative care needs, there is significant variability in timing and access to such services (Catriona R Mayland et al. 2021b). In addition, geographic disparities exist in the diagnosis of HNC: rural patients are significantly less likely to be diagnosed with HNC at an early stage than are urban patients (Mukherjee et al. 2020) and are less likely to have access to specialized oncologic care (Lowe and Nobriga 2021). This is especially important in HNC, where only one-third of patients present with early stage disease (Osazuwa-Peters et al. 2016; Penner 2009). Consequently, older patients with HNC in rural settings may be offered palliative and EOL care late in their disease trajectories (Sesterhenn et al. 2008). Multiple studies have demonstrated the profound benefits of early initiation of palliative care (Haun et al. 2017; Temel et al. 2010); an effective model for comfort care for HNC patients should emphasize both early detection of disease and early consideration of palliative measures in conjunction with treatment to maximize QOL for patients and their caregivers. In helping HNC patients weigh their treatment options, clinicians should take into account the socioeconomic, and other, factors that might otherwise prevent some HNC patients from initiating treatment and palliation at an earlier stage.

### EOL care in the acute setting

EOL care is a term that encapsulates care given in the final days to months of life. Acute EOL care refers to such measures taken in the acute setting, such as the emergency department or an inpatient environment. Acute EOL care strategies have had mixed results. It is possible for patients to view such care experiences in a positive light, especially if their acute needs can be met quickly (Robertson et al. 2021). However, acute EOL interventions have also been reported to be clouded by a preoccupation with treatment and a negative perception of palliative care, instead of an emphasis on patient comfort (Willard and Luker 2006).

Though most deaths in the developed world occur in the acute care setting, few are managed by palliative care providers (Phillips et al. 2011). The acute care environment often emphasizes curing disease and prolonging life, which can make the transition to palliation even more challenging (Gibbins et al. 2009). There is a paucity of specific research on acute EOL care for HNC patients, though these patients are likely to present to the hospital with concerns regarding breathing, pain, or cancer progression. Further studies might elucidate how acute EOL guidelines, such as formulating advance directives, might help these patients.

### Hospice and home care

Hospice care is a type of palliative care for patients with an estimated lifespan of 6 months or less; its use, by definition, necessitates the withdrawal of curative treatments (Shalev et al. 2018). Hospice care can be offered in a hospice facility or in the patient’s home; the mode of delivery of such services usually depends on patient and/or family preferences. Though HNC patients have significant disease-related morbidity that often requires complex management (for example, tracheostomy care), very few actually utilize hospice care. A 2019 study reported that only 3.5% of HNC patients enrolled in hospice, with 21.3% of them spending under 3 days in hospice, suggesting either the continuation of aggressive EOL treatment instead of comfort-based alleviation of symptoms (Chen et al. 2019) or simply a reluctance to engage in a treatment plan that does not aim for full cure.

HNC (especially laryngeal and hypopharyngeal cancer) patients are less likely to die at home or in hospice, as opposed to in the hospital (Stephens et al. 2020). This may reflect a tendency to treat the particularly morbid symptoms associated with this disease in an inpatient setting, as opposed to equipping patients and caregivers to manage these symptoms in a more comfortable, outpatient environment. Effective hospice care for these individuals would ideally address the disproportionate symptomatic burden these patients face and provide support around the anguish these symptoms cause for both the afflicted individuals and their loved ones.

A 2011 study found that HNC patients’ families reported better EOL care if patient death occurred at home or hospice instead of a hospital, or if a palliative team guided symptom management (Shuman et al. 2011). Palliation, especially the thoughtful use of home hospice, has a significant positive impact on treatment quality at the end of life; this is reflected in the ASCO updated recommendations that all patients with advanced cancer receive palliative care alongside usual oncology treatment (Smith et al. 2017). Adherence to these guidelines might help terminal HNC patients feel more satisfied with their EOL care.

### Recommendations for treatment teams to encourage palliative care initiation

The decision to initiate palliative measures in HNC patients often depends upon the qualities and dynamics of treatment teams. Providers who make pragmatic suggestions, normalize early conversations and education about palliation, and build trusting and honest relationships with patients might find greater success in engaging patients with palliative care. In addition, physicians might utilize non-curative treatment modalities to help patients find more comfort. The current literature on palliation contains many recommendations that physicians might use to facilitate the usage of this type of care; several of these suggestions are listed in Table 1.

Studies that examine how best to implement comfort-based care have consistently shown that early initiation of palliative care using an interdisciplinary approach can help maximize patient benefits (Schenker et al. 2016). Introducing these considerations early in the treatment course, with palliative care specialists working in conjunction with other care team members, can help normalize discussions about symptomatic burden and address potential issues before they grow to cause significant distress (Bonomo et al. 2019; Ferrell et al. 2017); one way to facilitate this partnership might be to involve palliative specialists regularly in tumor board meetings, where these patients are discussed. Furthermore, patients who consider EOL care early in their disease courses are less likely to receive needlessly painful care and are more likely to enroll in hospice when appropriate (Hoesseini et al. 2020). The normalization of palliative care use is especially important for its continued implementation; the decision to engage in palliative and hospice care is strongly linked to physician qualities, including the individual physician’s prior usage of hospice care for other patients (Obermeyer et al. 2015).

It is important that clinicians be realistic, educate patients about their options, and inform patients if there remains no effective treatment. This is especially crucial in HNC because of the multifaceted considerations that affect patient comfort (Sciubba 2009). Physician-documented goals of care discussions – including conversations about prognosis, treatments, and palliation – have been shown to correlate with comfort care utilization (Vukkadala et al. 2021). Further, educational materials tailored to health literacy levels can improve patients’ understanding of what post-treatment care might entail. Using the teach-back method might also help clinicians gauge patient understanding and reduce patient uncertainty (Ahmadidarrehsima et al. 2020). Providers should establish good communication with their patients to leverage the advantages of palliation. Studies have suggested that patients with HNC and their healthcare providers do not always communicate clearly: patients and professionals both have reported withholding key information in the clinical setting, which can be due to the environment of the encounter, a wish to portray a brave face, or an inability to recognize emotional distress (van den Heuvell et al. 2017). Providers should also remember that their own priorities might not match those of the individuals they are treating. Studies have shown that patients with HNC prioritize survival, pain management, expense coverage, and preservation of their abilities to perform activities of daily living (List et al. 2000; Tschiesner et al. 2013; Windon et al. 2019). However, these preferences vary among patients, and measuring them in aggregate may not reflect how any individual prioritizes treatment goals. For example, a 2011 study reported that, though interests often align, being pain-free and maintaining appearance were more important to treatment teams than to HNC patients themselves (Gill et al. 2011). Patients are more willing to take advantage of palliative treatment if they have trusting relationships with their providers (Salins et al. 2020); such trust flourishes when patients feel that they are understood.

In order for physicians to build this rapport, they must listen to and honor patient concerns and preferences while still providing informed insight. The current wisdom regarding palliative care emphasizes shared decision-making – the notion that patients, caregivers, and healthcare providers should all contribute to decisions regarding patient care. However, behavioral decision-making models have demonstrated that HNC patients often do not adhere to the typical algorithms that describe choices that other patients usually make. Instead, these patients, along with others coping with serious illness, considerable pain, and impaired ability to carry out activities of daily living, prefer to retain their roles as “ultimate decision makers” (approving treatment choices), but still look to providers to be “problem solvers” (proposing treatment options) (Davies et al. 2010). Therefore, effective comfort care for HNC patients might involve physicians suggesting palliative measures early in the illness course and dispelling myths about comfort care, while leaving space for patients themselves to approve or amend the proposed plan. Indeed, advanced cancer patients and their families have reported strong desires to contribute to decisions about treatment preferences (Steinhauser et al. 2000) – a wish that should be honored throughout the course of care.

Another way to facilitate conversations about EOL care is for physicians themselves to address their own discomfort with these difficult discussions. Clinicians often have a natural impulse to treat underlying conditions and strive for a cure instead of aiming for patient comfort; as a result, doctors can be reluctant to discuss HNC prognosis during what could otherwise be a meaningful palliative phase of treatment (Hoesseini et al. 2020). Physicians might also be reluctant to discuss prognosis in the palliative phase due to a fear of inaccuracy (Hoesseini et al. 2020). In addition, a 2020 study reported that physicians tend to overestimate HNC patients’ survival predictions, which were overly optimistic for 59% of patients, and accurate for only 18% (Hoesseini et al. 2020); the same study reported that HNC patients also tend to overestimate their own survival prospects. These inaccurate estimations have the potential to jeopardize care planning by causing treatment teams to maintain false hope for cure and impose painful interventions on patients to avoid uncomfortable conversations. More accurate prognostic models and the early introduction of palliative considerations in a sensitive but honest conversation about prognosis can help HNC patients optimize their final weeks.

Physicians can also alleviate the unease surrounding these discussions by dispelling the notion that initiating palliation or hospice is “giving up” on a patient. In actuality, the requirement for hospice initiation is the discontinuation of curative care (Medicare.gov) – far from abandoning the goals of patient comfort and satisfaction. No similar requirement exists for palliative care. The framing of information about these options affects whether patients choose to pursue palliation or aim for full recovery. Discontinuing curative treatment may induce feelings of uncertainty, and patients are often biased to continue a treatment they have started, regardless of whether completing the course will be beneficial (van den Heuvell et al. 2017). Palliation can be daunting when it is seen as a step that necessarily precedes death; early conversations about palliative care can destigmatize its use, and instead empower patients and families to be proactive in their management of HNC and ensure their wishes are respected (Shuman et al. 2011). Furthermore, HNC patients are unlikely to suggest transfer to a hospice institution independently, often because they are not aware that such resources exist (Sesterhenn et al. 2008).

It should also be noted that palliative chemotherapy is effective in some cases of recurrent or metastatic HNC (Rajendra et al. 2020). This chemotherapy might be delivered concurrently with radiotherapy (Kumar et al. 2015), though the benefits and drawbacks of different treatment regimens are understudied. Similarly, surgical palliation, including wound management and tumor debulking, may help alleviate symptoms that are otherwise challenging to treat (Chan et al. 2013). It is important that medical and surgical treatments be considered not only with curative intent, but also in the context of palliation.

### Identifying a good death

Patients who fail curative treatment and must face EOL decisions are appropriate candidates for hospice care and discussions about death. In these conversations, providers and patients must consider factors that might make a patient’s death more dignified and peaceful.

The modern hospice movement has redefined a “good death” as one that includes acceptance and closure, symptom management, and a sense of completion (Steinhauser et al. 2000), instead of one that indiscriminately prolongs survival. In pursuit of this goal, it is important that healthcare providers listen to patient priorities and concerns without making assumptions. HNC patients are often viewed as socially disadvantaged and vulnerable, which can spur doctors to adopt paternalistic attitudes that might preclude them from otherwise honoring personalized treatment decisions (van den Heuvell et al. 2017). HNC patients are often not proactive about voicing their preferences, and commonly delay seeking treatment due to shame or unawareness of their disease severity (van den Heuvell et al. 2017), This is a potential avenue for improvement in enhancing comfort-based care for HNC patients.

These palliative services need not be provided solely in the hospital: comfort care in an outpatient setting can allow patients to die surrounded by loved ones at home, which is what these individuals frequently prefer – though this often does not happen (Townsend et al. 1990). Instead, these individuals commonly die with poor symptom control in an unfamiliar environment (Ellershaw et al. 2003). It is clear that there is often a mismatch between a theoretical “ideal death” and the death that actually occurs, and a similar discrepancy is apparent in healthcare policy: guidelines from the National Comprehensive Cancer Network lack discussion of palliative care measures in oncology, despite the advantages that standardization and widespread utilization of clinical palliative practice might have for cancer patients (Howie and Peppercorn 2013; Sullivan et al. 2019; Thompson et al. 2021), especially for individuals with HNC.

Though few studies have quantified the number of cancer patients who consider their dying environments to be “dignified,” several qualitative investigations have sought to identify aspects of a “dignified death” (Chochinov et al. 2002; Enes 2003; Pleschberger 2007; Rodríguez-Prat et al. 2016), suggesting a growing interest in providing this outcome for terminally ill patients. Retrospective analyses have suggested that, while care teams often address biomedical aspects of HNC care, psychosocial and spiritual components are often not documented; considering preferences beyond those related strictly to medical care might help physicians facilitate the coveted balance between a fulfilling life and a dignified death. While palliative measures exist on a spectrum ranging from minor changes for symptom relief to complete discontinuation of curative care, all aim toward the common goal of helping patients achieve a “good death”; this has the potential to be especially meaningful for HNC patients, who are particularly likely to benefit from comfort care.

## Conclusion

Palliative and hospice care are intrinsically difficult topics to discuss. These conversations involve consideration of impending death and can be distressing for patients and their loved ones. HNC patients have significant symptom burdens and are especially likely to benefit from the early incorporation of palliative treatments; however, these patients often do not access these care options due to several aspects of their illnesses, treatments, and psychosocial situations. The paradigm of palliative care has shifted toward a framework that emphasizes patient comfort without sacrificing disease treatment, but there is room for improvement, especially for HNC patients. Physician training in conversations about comfort care, normalization of palliation as a standard component of treatment, and HNC-specific considerations in palliative care all have the potential to maximize patient and caregiver satisfaction with non-curative treatment.

## Figures and Tables

**Table 1.** Recommendations for palliative care based on current literature

Study	Recommendation
Bauman and El-Jawahri 2021	Palliative HNC care in different populations should consider the unique needs of different groups.
Chou et al. 2021	Hospice care can reduce the use of invasive medical interventions in patients with terminal head and neck cancer and may improve their quality of death. Hospice care enrollment for over 3 months can save unnecessary medical expenditures for these patients.
Vukkadala et al. 2021	Better provider documentation of treatment directives and the reduction of low-utility treatment can improve end-of-life outcomes in HNC, as assessed by several measures.
Hoesseini et al. 2020	More accurate prognostic models can help physicians with personalized prognostic counseling of HNC patients.
Rajendra et al. 2020	Specific palliative single-agent or combination chemotherapy regimens can help maintain quality of life in HNC.
van den Heuvell et al. 2017	Tailored and personalized information specific to individual circumstances, preferences, and concerns should be implemented into the presentation of information to HNC to emphasize shared decision-making.
Schenker et al. 2016	Palliative care should be included as a standard component of the multidisciplinary treatment approach for HNSCC.
Kumar et al. 2015	Concurrent low-dose palliative chemotherapy plus radiotherapy in advanced HNC can improve quality of life.
Chan et al. 2013	Surgical palliation, including tumor debulking and wound management, is a good option for HNC patients with challenging pain symptoms.
Shuman et al. 2011	Patient death at home or in hospice care, as well as a palliative team guiding symptom management, all improve the end-of-life experience for HNC patients, as reported by patient families.
Sciubba 2009	Realistic communication of the status of HNC patient health is crucial to consideration of palliative management.
Sesterhenn et al. 2008	Physicians should talk to terminal HNC patients about the status and incurability of their disease as early as possible to aid patient and family decision-making.
